# Context of substance initiation among urban Native Americans: an exploratory retrospective case-control study

**DOI:** 10.7717/peerj.16482

**Published:** 2023-11-27

**Authors:** Nicholas Guenzel, Hongying Daisy Dai, Lyndsay Dean

**Affiliations:** 1 College of Nursing, University of Nebraska Medical Center, Lincoln, NE, United States of America; 2 College of Public Health, University of Nebraska Medical Center, Omaha, NE, United States of America; 3 College of Nursing, University of Nebraska Medical Center, Omaha, NE, United States of America

**Keywords:** Native American, Addiction, Urban

## Abstract

**Background:**

Addiction is a significant problem among many Native American groups but has rarely been examined in urban populations. In particular, little is known about the context in which urban Native Americans first use substances. This study compares cases (people with a history of addiction) to controls (people without a history of addiction) on demographics, substance use history, context of first substance use, and polysubstance use. In addition, this appears to be the first study to overcome the lack of Native American professionals by employing and training lay community members to identify criteria of substance use disorders in survey participants. Employing community members helped foster trust that enabled the revelation of sensitive and often illegal activity. As a result, the investigators were able to recruit participants who likely would not have engaged with traditional researchers.

**Methods:**

The trained Native American lay research assistants recruited community members and administered surveys. They first asked questions regarding the criteria for substance use disorders. Individuals who were determined to have met criteria for a substance use disorder in the past were classified as cases (*n* = 38) and those who never met such criteria were classified as controls (*n* = 42). They then asked demographic, substance use, and polysubstance use questions. Lastly, eight cases and eight controls were randomly selected for a second interview by a licensed drug and alcohol counselor (LDAC) who conducted a blinded assessment regarding the presence or absence of a history of a substance use disorder.

**Results:**

Both groups reported a relatively young age of first substance use (age 16 years for cases and age 15 years for controls). Alcohol was the first substance most commonly used in both groups. Controls reported first benzodiazepine use at a younger age than cases but no other significant differences were found. Both groups reported first obtaining their first drug from family, friends, or at home (rather than a party, bar, or store). Most commonly, the location of their first use of drugs occurred at a friend’s home, a party, a bar, or school rather than at their own home. Cases were marginally more likely to report that their first drug use occurred with a friend rather than with a family member when compared with controls. The majority of both groups reported that their first drug use occurred with other Native Americans rather than with non-Native Americans. Polysubstance use was common in both groups (43–45%). There were no significant differences between the groups regarding polysubstance use. The LDAC arrived at the same determination as the trained research assistants on all eight cases and eight controls.

## Introduction

Native Americans often have substance use disorders at higher rates than any other ethnic group, threatening the very existence of many communities across the country. Recent data show that Native Americans have rates of binge drinking that are second only to people who identify as multiracial. Native Americans also have the highest rates of illicit substance use, substance use disorders, and people in need of substance use treatment (Center for Behavioral Health Statistics Quality, 2022). This group also has the highest rate of past year substance use disorder at 11.2% compared to Whites (7.8%), African Americans (7.1%), Hispanics (7.1%), and Asians (4.1%) (Statistics C for BH and Quality., 2021). In addition, some evidence suggested that Native Americans have a younger age of first use, an earlier age of substance use disorders, and are more likely to have a multi-substance use disorder than many other populations, all of which are associated with a worse prognosis (Dickerson et al., 2012; Gilder et al., 2016; Henry et al., 2011).

Another factor that magnifies the impact of substance use among Native Americans is that they have some of the highest rates of diabetes among any other racial group. One national estimate indicated that 23.5% of Native Americans, 18 or older, have diabetes which is 2.9 times higher than non-Hispanic Whites (Centers for Disease Control and Prevention, 2017). Researchers have found evidence that substance use should be considered to be a risk factor for new-onset diabetes (Pastor et al., 2020). In addition, substance use among people with diabetes has been associated diabetes-related complications and mortality (Winhusen et al., 2019). We have a deficit of knowledge about urban Native Americans in the upper Midwest region which impairs the development of effective prevention and treatment interventions. Most studies with Native Americans have either been completed in other regions (Akins et al., 2013; Copeland et al., 2017; Friese et al., 2011; Stanley et al., 2009) or been conducted on reservations (Armenta, Sittner & Whitbeck, 2016; Armenta, Sittner & Whitbeck, 2016; Armenta, Sittner & Whitbeck, 2016; D’Amico et al., 2021a; D’Amico et al., 2021; Miller, Stanley & Beauvais, 2012; O’Connell et al., 2011; Whitesell et al., 2007). There is evidence that substance use among Native Americans is higher in the Midwest region and also differs significantly between reservations and urban areas (Miller, Stanley & Beauvais, 2012).

Working with minoritized populations presents significant challenges and lay individuals have shown promise in helping with mental health and addiction issues (Mutamba et al., 2013). One large trial showed that lay counselors were helpful in screening and providing brief interventions for depression (Patel et al., 2017). Such projects appear to be more active in low- and middle-income countries where healthcare providers may be difficult to access (Mutamba et al., 2013). Successful projects have engaged lay Native Americans in a variety of health targets including suicide and substance use screening (Hopson et al., 2022; Kegler et al., 2003; Watts et al., 2005). Almost all identified studies have used lay individuals for screening rather than the inference of meeting criteria for a specific diagnosis.

Few studies have examined critical contextual factors surrounding substance initiation. Currently, only one study has identified found that Native Americans were more likely to get substances through social sources or theft (Friese et al., 2011). This study did not examine the ethnicity of the individuals with whom the participants first used illicit substances or other factors influencing their first use. A number of articles have examined programs used to facilitate addiction recovery among Native Americans, including drumming therapy (Dickerson et al., 2021), Wellbriety (Moore & Coyhis, 2010), the Red Road (Gone, 2011), and non-Native specific programs such as Alcoholics Anonymous (Muñoz & Tonigan, 2017). However, no identified studies have examined using naturalistic methods, the ways in which Native Americans have succeeded in recovering from addiction in the past, including the number of times they have tried to quit, the use of Native traditions, and other methods (*i.e.,* Alcoholics Anonymous, inpatient treatment, outpatient treatment).

There is a clear deficit in research on addiction in urban Native American populations. However, given abuses both in research and in society at large, members of these communities may be understandably reluctant to engage with researchers. Furthermore, the current study involves disclosing illegal activities, likely further contributing to resistance. Native Americans are underrepresented in the healthcare professions, so may be unavailable for research trials. To help overcome this barrier, we employed Native American community members and trained them to determine whether or not a participant likely met the criteria for a substance use disorder based on the Diagnostic and Statistical Manual for Mental Disorders criteria. As trusted members of the community, these individuals then recruited all of the participants and administered the surveys.

In this study, we recruited urban Native Americans for a cross-sectional survey. Individuals who were determined to have met criteria for a substance use disorder in the past served as “cases.” Participants who had used a substance at least once but who had likely never met the criteria for a substance use disorder served as “control.” Individuals who had never used a substance or who were determined to currently meet criteria for a substance use disorder were excluded. Survey questions elicited information about the context of first substance used, demographics, and polysubstance use.

The aims of this study were to:

1. Compare the demographics of cases to controls including age, sex, history of living on a reservation or in foster care, and having a first-degree relative with addiction issues.
2. Compare ever and first substance use between cases and controls including age, source, place, and social context of first use.
3. Examine reported polysubstance use among and between cases and controls.
4. Examine the ability of lay individuals to determine the presence or absence of a history of a substance use disorder.

## Literature Review

Substance use within the Native American population is a widely known issue but the underlying causes are often falsely assumed or gravely misinterpreted by the health care system (Bingham & Kelley, 2022; Mancuso, 2018; Wimbish-Cirilo et al., 2020). Currently, the Western mental health care models are most often derived from the general population and cannot be effectively applied to other diverse cultures (Bingham & Kelley, 2022). For instance, a prime example is the common use of Western standardized screening tools to assess and/or evaluate the recovery process. These instruments lack cultural context and have biases, especially when considering non-White populations (Bingham & Kelley, 2022). As a result, this leads to inadequate treatment approaches that constantly miss target outcomes along with causing an economic burden to the nation’s communities (Mancuso, 2018; Wimbish-Cirilo et al., 2020).

Historical trauma is considered to be a primary root cause for substance misuse issues experienced by Native Americans (Craig Rushing et al., 2021; Mancuso, 2018; Wimbish-Cirilo et al., 2020). For instance, past accounts provide a clear perspective on how early European settlers profoundly jeopardized the Native Americans’ peaceful way of life. In the early 1600s, the Europeans’ arrival to the United States contributed to the onset of many deadly diseases, unnecessary violent assaults, cultural genocide, forced assimilation, and mandatory migration within the Native American population (D’Amico et al., 2021a; D’Amico et al., 2021; Mancuso, 2018; Myhra, 2011). In addition, historical documentation further depicts how the Europeans used alcohol, otherwise known as “firewater,” with the intent to manipulate and make the Native Americans vulnerable during trade negotiations (Mancuso, 2018). Even though some Native Americans recognized the dangers of alcohol, this substance continued to be frequently traded within native tribes, along with the trading of other addictive substances leading to ongoing substance misuse issues that were passed along to subsequent generations (D’Amico et al., 2021a; D’Amico et al., 2021; Mancuso, 2018; Mignon & Holmes, 2013). Although the long-ranging consequences could not have been known at the time, it is now clear that addictive substances serve as the gateway for adverse behaviors including domestic violence, unstable caregiving patterns, and the use of additional dangerous substances (Brockie et al., 2015; Stanley et al., 2020a; Stanley et al., 2020b). Consequently, these behaviors initiate a recurring cycle of intergenerational trauma and substance misuse within the Native American population. This concept is especially true when understanding the onset and progression of certain risk factors for Native American youth.

In the United States, approximately 1.6 million youth (less than 18 years of age) identify as Native American or Alaska Native (Craig Rushing et al., 2021). Even though this population exudes great resilience and strength, they are disproportionately affected by mental health issues compounded by addiction (Brockie et al., 2015; Craig Rushing et al., 2021; Currie et al., 2013; Stanley et al., 2020a; Stanley et al., 2020b). Research has shown a rise in challenges surrounding substance misuse within the Native American youth including younger ages of substance use and/or the misuse of addictive substances (Brockie et al., 2015; Kopak, Proctor & Hoffmann, 2017; Stanley et al., 2020a; Stanley et al., 2020b).

Native Americans often have a younger age of substance use initiation when compared with other racial groups. Native Americans are second only to the multiple-race individuals in having the youngest age of first cannabis use. Native Americans have the youngest age of first alcohol use and the youngest median age of substance use initiation overall (Clemans-Cope et al., 2022). Research has shown that individuals who use alcohol and marijuana before age 14 years have a greater lifetime risk for prescription drug misuse compared with peers who started substance use at an older age (Stanley et al., 2020a; Stanley et al., 2020b). Additionally, youth who used substances at an early age, had a greater risk of consuming alcohol along with other drugs, including prescription pain medications (Stanley et al., 2020a; Stanley et al., 2020b). One study of a Midwestern reservation found that over 50% of participants between the ages of 18 and 25 years, reported opioid misuse (Momper et al., 2013). Moreover, studies have shown that Native American adolescents who resided on or near reservations, experimented with alcohol, marijuana, and inhalants at an earlier age when compared to Caucasian adolescents who had the same living conditions (Spillane et al., 2020; Stanley et al., 2020a; Stanley et al., 2020b). For instance, Native American youth were over three times as likely to be classified as early users of marijuana (35.9%) when compared to non-Native American youth (11.6%) (Stanley et al., 2020a; Stanley et al., 2020b).

Research has also shown that Native American youth are at an elevated risk for substance use problems when they were raised by a caregiver who misused substances (Kopak, Proctor & Hoffmann, 2017; Mancuso, 2018; Mignon & Holmes, 2013). There is strong evidence that unhealthy caregiving styles and/or adverse interactions that occur early in childhood are often linked to substance use, other mental health disorders, and suicide attempts in youth (Geoffroy, Gunnell & Power, 2014). Parental stress, addiction issues, maladaptive caregiver-child attachments, multigenerational hardships, and minimal or absent oversight of the child by caregivers also contribute to youth substance misuse (Geoffroy, Gunnell & Power, 2014). In addition, it has been found that Native Americans have the highest rate for Adverse Childhood Experiences (ACEs) when compared to any other ethnic group (McDonnell & Valentino, 2016). ACEs are well-known to negatively impact the caregivers’ ability to effectively raise children since they are often associated with depression, substance misuse, suicide, and harmful discipline practices, such as corporal punishment towards their young (Geoffroy, Gunnell & Power, 2014; Mignon & Holmes, 2013). Therefore, research clearly depicts how family violence is tightly interwoven with substance misuse, often contributing to the onset of addiction issues within youth (Geoffroy, Gunnell & Power, 2014; Kopak, Proctor & Hoffmann, 2017; Mignon & Holmes, 2013). On the other hand, a buffer from the risks associated with substance use is enhanced when youth are surrounded by protective social factors, such as a sense of belonging, acceptance, and unconditional love within their family unit and/or peer system (Craig Rushing et al., 2021; Kopak, Proctor & Hoffmann, 2017). These findings also suggest that a strong identity that embraces cultural pride helps build resiliency against substance use along with achieving better health outcomes in Native American youth (Brown, Dickerson & D’Amico, 2016; Craig Rushing et al., 2021).

Emerging research signifies how substance misuse issues are considered to be a multi-dimensional disorder that adversely impacts many facets of one’s wellbeing (*i.e.,* physical, mental/emotional, and spiritual health) (Brown, Dickerson & D’Amico, 2016; D’Amico et al., 2021a; D’Amico et al., 2021; Spillane et al., 2020). Consequently, it is imperative for society to embrace nonjudgmental approaches that best meet the health care needs of Native Americans (D’Amico et al., 2021a; D’Amico et al., 2021; Lowe et al., 2012). Currently, there is a paucity of research on addiction among urban Native Americans (D’Amico et al., 2021a; D’Amico et al., 2021; Kopak, Proctor & Hoffmann, 2017; Wimbish-Cirilo et al., 2020). Finally, more research is needed to thoroughly examine the treatment barriers, including health inequities and disparities, along with discovering innovative ways to optimize treatment engagement for Native American youth (Boyd et al., 2021).

## Materials & Methods

### Design

A retrospective cross-sectional case-control design with a community-based convenience sample was employed. “Cases” were individuals who had a history of addiction while “controls” were those without a history of addiction. The University of Nebraska Institutional Review Board approved of this research (386-18-FB).

### Methods

Two female Native American research assistants were recruited based on their level of activity in the community, a history of working with non-clinical supportive mental health services, and successful past research collaboration. One is from a tribe in Nebraska and was raised in an urban setting. The other was raised on a reservation in South Dakota. Both research assistants have had individuals close to them who have struggled with addiction and received various forms of mental health treatment. This background likely gave them some insight in this area and facilitated their connection with participants. The PI (a psychiatric nurse practitioner) trained the two research assistants to assess for substance use disorders. He reviewed the DSM criteria of the disorders and the various ways these criteria can manifest in diverse individuals. He also gave examples of common questions used to assess for these criteria. The PI also gave the research assistants videos discussing the assessment and diagnosis of substance use disorders. He then assessed their knowledge by having them teach back the material to ensure competence in correctly differentiating individuals who likely met criteria for a substance use disorder in the past *versus* those who likely did not. He allowed them to practice assessments and provided constructive feedback when needed. Lastly, he answered questions and was available for ongoing consultation. The research assistants were also trained in the use of use of the REDCap system.

Research assistants contacted community members they knew who may have met inclusion criteria and invited them to participate. Additional potential participants learned of the study by word-of-mouth and contacted one of the research assistants. The research assistant ensured individuals meet the inclusion criteria by verifying that individuals who would be in either group: (1) were 19 years of age or older, (2) self-identified as Native American, (3) resided within 30 miles of Lincoln or Omaha, (4) had used a drug or alcohol at least once in their lives, and (5) would not have likely meet criteria for a substance use disorder at the present time. Individuals with ongoing problems with drugs or alcohol were told that they do not qualify for this study and were given a list of local treatment resources.

Individuals who wished to participate were given information about the study. The interviewer ensured that the participant understood all aspects of the study before giving them the opportunity to sign the consent in-person or mailed it to the PI. The participant then answered the survey questions over the phone or in person. The research assistant recorded the survey responses in REDCap which is an internet-based research data collection system. Participants were given a Visa card for their time.

The research assistants asked eleven questions corresponding to the DSM criteria for substance use disorders. This more flexible approach was preferred to the standardized tool due to the significant diversity in the population. The research assistants could modify the language in ways that did not change the meaning for participants who had less than a high school education *versus* a participant who had a college degrees, for example. After each positive response, the research assistant asked the participant if they were currently having the issue or had this issue in the past (*i.e.,* if they used more than intended in the past or currently). After the initial questions, the research assistants determined if the participant met two or more of the criteria within a twelve-month period.

Participants were then placed in two groups: those who likely met criteria for a substance use disorder in the past (cases, *n* = 42) and those who likely never met criteria for a substance use disorder other than tobacco (controls, *n* = 38). All participants were asked questions on demographics, tobacco, all substances ever used, and substance history questions. Cases completed two additional sections including identifying problematic substances and their history with those problematic substances.

The surveys took an average of approximately 30 minutes each. When surveys were completed in person, they most commonly took place in a private location in the participant’s or research assistant’s home. For individuals who were determined to have had a history of addiction, an additional two minutes were spent on each problematic substance. Questions included age at which the substance became a problem, how often, and in what quantity they used. All individuals were asked at what age they first used each substance they tried, who they first used with (and whether they were Native American or non-Native American), where they first used, and where they obtained the substance. Individuals who completed a second assessment with the Licensed Drug and Alcohol Counselor (LDAC) received a second Visa card for their time. The original data collection occurred between February and October 2019 and the LDAC assessments occurred in November and December 2019.

Within two months of completion of all surveys, eight disorder group participants and eight control group participants were randomly selected for a follow-up assessment by a LDAC to confirm the presence or absence of a history of substance problems. The counselor completed assessments over the phone and was blinded as to the original determination of the participants’ substance history.

## Results

Aim 1: Compare the demographics of cases (*n* = 42) to controls (*n* = 38) including age, sex, history of living on a reservation or in foster care, and having a first-degree relative with addiction

There were no significant differences between cases and control on any demographic factors including age, sex, history of living on a reservation or in foster care, having a first-degree relative who likely has a substance use disorder, or current daily tobacco use (see Table 1).

**Table 1:** Demographics and tobacco use.

	Cases	Controls	*P*-value
N	38	42	
Age (Mean (SD))	40.3 (13.1)	40.4 (14.4)	*p* = 0.97^†^
Female (n, percentage)	27 (71.1%)	27 (64.3%)	*p* = 0.56^#^
Years on reservation (Mean (SD))	9.6 (7.5)	6 (5)	*p* = 0.11^†^
Ever lived on reservation (n, percentage)	23 (60.5%)	19 (45.2%)	*p* = 0.17^#^
Years on reservation before age 19 (Mean (SD))	7.2 (7.2)	7.1 (5.3)	*p* = 0.97^†^
Ever in foster care (n, percentage)	10 (26.3%)	15 (36.6%)	*p* = 0.33^#^
Years in foster care (Mean (SD))	4.1 (4.9)	5.9 (5.1)	*p* = 0.42^†^
First-degree relative with addiction (n, percentage)	31 (81.6%)	37 (88.1%)	*p* = 0.42^#^
Ever daily tobacco use (n, percentage)	22 (58%)	23 (54.8%)	*p* = 0.78^#^
Age of first tobacco use (Mean (SD))	14.5 (3.8)	13.4 (4.4)	*p* = 0.39^†^
Current daily tobacco use (n, percentage)	14 (63.6%)	15 (65.2%)	*p* = 0.91^#^

**Notes.**

† Independent samples T-Test.

# Chi-square test.

Aim 2: Compare ever and first substance use between cases and controls including age, source, place, and social context of first use

Participants reported a mean age of first substance use of 16 years for cases and 15 years for controls. In both groups, alcohol and cannabis were both first used at a mean age of 15–16 years while the other substances were commonly first used between ages 19 and 29 years. Controls reported first using benzodiazepines at a significantly earlier age than cases. There were no other significant differences of age at first use. In both groups, alcohol was the drug most participants used first.

Both cases and controls reported obtaining the first drug they used from family, friends, at home, or a spouse rather than a party, bar, or store (76.3% and 82.5%, respectively). However, the majority of both groups reported using their first drug at a friend’s home, party, or school rather than at home. Cases were marginally more likely to report that their first use occurred with a friend than a family member compared with controls. Both cases and control reported that their first use occurred with Native Americans rather than people of other races (56.8% *v.* 68.3%).

Most participants reported using alcohol at least once, approximately 40% of both groups reported ever using cannabis, and approximately 10–20% of both groups reported using other substances (see Table 2). There were no significant differences in the proportion of each group who reported using each substance or the number of types of substances used.

**Table 2:** Age, substance, and context of first drug use.

	Cases	Controls	
N	38	42	
Age of first use:			
Alcohol (Mean (SD))	15.8 (2.9)	15.2 (4.6)	*p* = 0.47^†^
Cannabis (Mean (SD))	15.5 (3.4)	15 (4)	*p* = 0.69^†^
Methamphetamines (Mean (SD))	28.6 (7.4)	25.4 (4.1)	*p* = 0.29^†^
Crack (Mean (SD))	21.7 (6.7)	19.9 (4.3)	*p* = 0.61^†^
Opioids (Mean (SD))	21.7 (3.1)	21.8 (8)	*p* = 0.97^†^
Benzodiazepines (Mean (SD))	27 (1.7)	19 (4.6)	*p* = 0.047^†^^*^
Number of drugs ever used (Mean (SD))	1.76 (1.1)	1.93 (1.2)	*p* = 0.54^†^
First substance age (Mean (SD))	16 (3.5)	15 (4.3)	*p* = 0.13^†^
Ever used:			
Alcohol (n, percentage)	37 (97.4%)	37 (88.1%)	*p* = 0.12^#^
Cannabis (n, percentage)	15 (39.5%)	18 (42.9%)	*p* = 0.76^#^
Methamphetamines (n, percentage)	5 (13.2%)	10 (23.8%)	*p* = 0.22^#^
Crack/cocaine (n, percentage)	3 (7.9%)	7 (16.7%)	*p* = 0.24^#^
Opioids (n, percentage)	4 (10.5%)	6 (14.3%)	*p* = 0.61^#^
Benzodiazepines (n, percentage)	3 (7.9%)	3 (7.1%)	*p* = 0.89^#^
First substance ever used: Alcohol (v. other drug) (n, percentage)	29 (76.3%)	26 (61.9%)	*p* = 0.17^#^
Source of first drug (family/friends/home/spouse v. party/bar/store) (n, percentage)	32 (86.5%)	33 (82.5%)	*p* = 0.63^#^
Location of first drug use (home/family v. friends/party/school) (n, percentage)	12 (32.4%)	16 (41%)	*p* = 0.44^#^
Social context of first drug (family/partner v. family) (n, percentage)	14 (40%)	23 (60.5%)	*p* = 0.08^#^
First used drug with another Native (v. non-Native) (n, percentage)	21 (56.8%)	28 (68.3%)	*p* = 0.29^#^

**Notes.**

† Independent samples T-Test.

# Chi-square test.

* *p* < 0.05.

Aim 3: Examine the reported polysubstance use among and between cases and controls

Polysubstance use was common in both groups with 44.7% of cases and 42.9% of controls ever using more than one substance at the same time (see Table 3). There were no significant differences between the groups in the likelihood of reporting polysubstance use or the number of combinations used. Alcohol/cannabis were frequently reported as being used in combination. In fact, 86% of individuals who reported using each substance reported using them together at least once. The combination of alcohol and methamphetamines was also common (79% of people who reported ever using both substances). Alcohol and crack were tried together by 70% of individuals who reported ever using both substances. Cannabis and methamphetamines were used together by 25% of people who reported ever using both substances. All other substance combinations were used by fewer than ten participants.

**Table 3:** Polydrug use by group.

	Cases	Controls	
N	38	42	
Have ever used two or more drugs at the same time (n, percentage)	17 (44.7%)	18 (42.9%)	*p* = 0.87^#^
Number of combinations of drug use (Mean (SD))	0.9 (2.2)	0.8 (1.2)	*p* = 0.78^†^

**Notes.**

† Independent samples T-Test.

# Chi-square test.

Aim 4: Examine the ability of lay individuals to determine the presence or absence of a history of a substance use disorder

The LDAC arrived at the same assessment as the community research assistants on all eight cases and eight controls. We were unable to compare the severity of the past disorder as the research assistants were not trained at this level of assessment. In addition, the accuracy of recalling concurrent symptoms that may have occurred years ago would have been questionable.

## Discussion

Addiction among Native Americans has long been a significant problem on many reservations (Soto et al., 2022). However, less research has examined substance use problems among the majority of Native Americans who now live off reservations (D’Amico et al., 2021; Kopak, Proctor & Hoffmann, 2017; Wimbish-Cirilo et al., 2020). This lack of information makes it difficult to develop effective programs to prevent or delay substance initiation, assess for risky substance use patterns, or address specific AI needs for recovery support.

This analysis had a number of limitations. This exploratory analysis was clearly underpowered to conclude that significant differences in demographic factors (*e.g.*, history of living on a reservation, being in foster care, *etc.*) do not exist between individuals with a history of a substance use disorder and those without such a disorder. The design was retrospective, requiring some individuals to recall symptoms that may have occurred years earlier.

Most participants in both groups reported first getting their substance of first use from family/friends/home/spouse rather than at a party/bar/store. By contrast, most individuals in both groups reported first using substances at a friend’s home/party/school rather than at home. Both groups were roughly evenly divided in reporting that their first use occurred with family/partner *versus* a friend. Over half of participants in both groups reported first using with one or more other Native Americans rather non-Native Americans. Further research exploring this factor may help lay the foundation for home and family-based substance initiation prevention.

Polysubstance use was common with over 40% of both groups reporting using multiple substances at once. The most common combinations were alcohol and cannabis, but the majority of people who had also used alcohol and methamphetamines had used them together as well as alcohol and crack. The increasing risks of polysubstance use in recent years must be recognized now that many drug supplies contain contaminants, such as fentanyl, which may be unknown to the user.

Few significant differences were found between the two groups. The lack of significant differences may be due, in part, to the small sample size and the diversity in the sample. For example, some factors may be significant for individuals who spent much of their early life on reservations but not those raised in urban areas. This would result in an even smaller effective sample size to evaluate specific factors. A larger sample size may reveal significant differences between the groups. However, given the lack of trend towards significance, it is also possible that the identified factors do not differ between urban Native Americans with and without a history of a substance use disorder.

This was the first identified study to employ lay individuals trained to assess for a likely history of a substance use disorder. The use of lay people rather than professionals clearly has limitations, but also significant advantages in a study such as this one. People of minority groups who have endured a long history of discrimination in society in general and abuses in research, in particular may be reluctant to disclose sensitive information. This apprehension would likely be magnified in discussing illegal activity. The success of trained lay individuals in this study may provide support for a new method to study challenges within hard-to-reach populations.

The current study can contribute to the growing focus on substance use prevention and treatment for urban Native Americans (Dickerson et al., 2022). In particular, greater attention is being paid to the role Native American identity and traditional practices can play in addition recovery treatment (Dickerson et al., 2021). A growing body of work has examined how efforts for rural reservations compare to those for urban Native Americans (Komro et al., 2022). Lastly, following a trend for more focus on the influence of social networks, more researchers are examining how social networks among urban Native Americans impacts the risk for addiction (D’Amico et al., 2021a; D’Amico et al., 2021). Retrospective analyses of the social context of first substance use, such as in this study, can help lay the foundations for further prevention work.

## Conclusions

Little research has been completed on urban Native American substance use. A better understanding of substance initiation in this population could help inform interventions for the prevention and early intervention for people in need. This study provides some insights into a Midwestern sample. It is notable that the mean age of first substance use was young in both groups. The majority of both groups reported obtaining their first substance from family, friend, at home or from a spouse. However, the majority of both groups first used the substance at a friend’s home, party, or school rather than at home. Just under half of both groups have engaged in polysubstance use which could add significantly to the risks of use depending on the particular substances used. One of the most significant contributions this study makes is demonstrating that lay individuals can be trained to conduct interviews in which they can reliably determine the presence of absence of a substance use disorder. Research assistants who are widely trusted within the community could help expand the understanding of substance use among individuals who have been marginalized and may have had challenging interactions with law enforcement and healthcare providers.

Future research could focus on larger sample sizes that may be more representative of the overall urban AI population and possibly reveal significant factors. A more in-depth social and substance use history could also provide greater insights into the development of addiction in this population. Lastly, a mixed methods approach with focus groups guiding the research questions could be helpful.

##  Supplemental Information

10.7717/peerj.16482/supp-1 Supplemental Information 1 Onset dataset

## Additional Information and Declarations

### Data Availability

The following information was supplied regarding data availability: The raw data is available in the Supplemental File.
